# Lower Limb Function in Elderly Korean Adults Is Related to Cognitive Function

**DOI:** 10.3390/jcm7050099

**Published:** 2018-05-01

**Authors:** A-Sol Kim, Hae-Jin Ko

**Affiliations:** 1 Department of Family Medicine, School of Medicine, Kyungpook National University, Kyungpook National University Chilgok Hospital, Daegu 41404, Korea; deepai@knu.ac.kr; 2 Department of Family Medicine, School of Medicine, Kyungpook National University, Kyungpook National University Hospital, Daegu 41944, Korea

**Keywords:** elderly, cognitive function, walking speed, postural balance

## Abstract

Patients with cognitive impairment have decreased lower limb function. Therefore, we aimed to investigate the relationship between lower limb function and cognitive disorders to determine whether lower limb function can be screened to identify cognitive decline. Using Korean National Health Insurance Service-National Sample Cohort database data, we assessed the cognitive and lower limb functioning of 66-year-olds who underwent national health screening between 2010 and 2014. Cognitive function was assessed via a questionnaire. Timed Up-and-Go (TUG) and one-leg-standing (OLS) tests were performed to evaluate lower limb function. Associations between cognitive and lower limb functions were analyzed, and optimal cut-off points for these tests to screen for cognitive decline, were determined. Cognitive function was significantly correlated with TUG interval (*r* = 0.414, *p* < 0.001) and OLS duration (*r* = −0.237, *p* < 0.001). Optimal cut-off points for screening cognitive disorders were >11 s and ≤12 s for TUG interval and OLS duration, respectively. Among 66-year-olds who underwent national health screening, a significant correlation between lower limb and cognitive function was demonstrated. The TUG and OLS tests are useful screening tools for cognitive disorders in elderly patients. A large-scale prospective cohort study should be conducted to investigate the causal relationship between cognitive and lower limb function.

## 1. Introduction

Generally, as age increases, lower limb function decreases (resulting in, for example, reduced walking speed and difficulty in balancing) and the risk of gait disturbance increases [1]. However, aging alone is not always directly linked to gait disturbance. Hence, there is active research on walking in the elderly. In particular, much research has been conducted to investigate gait disturbance as a predictor of cognitive disorders [2]. Normal walking is made possible through interactions between the sensory and motor systems. The executive control dimension, which plans an appropriate path and maintains safety while walking, decides on motor activity, the cognitive dimension is responsible for spatial perception and attention, and the affective dimension is involved in mood and a spirit of adventure [3]. Hence, the process of walking is related to high-level cognitive functioning. If cognitive function is impaired, gait disturbance, including abnormal walking speed, may occur [4].

Postural control is a crucial component of maintaining the balance of the body, and refers to appropriately controlling body posture to maintain the equilibrium and balance of the body in space [5]. Although postural control is conventionally regarded as an autonomic or reflexive task requiring minimal attention, numerous studies have demonstrated that it requires much attention depending on factors such as an individual’s age and balance function [6]. Thus, if attention is impaired due to decreased cognitive function, it may be difficult to control body posture, thus impairing balance function.

Patients with Alzheimer’s disease show diverse patterns of gait disturbance, including reduced walking speed, difficulty in initiating walking, unstable gait, and gait apraxia if severe [7]. Thus, elderly persons with no or mild cognitive impairment who exhibit mild memory loss but are not impaired in performing other daily activities should be approached with the serious recognition that lower limb function and gait ability may be predictors of cognitive disorder; additional research is needed to investigate the issue.

The Korean National Health Insurance Corporation (KNHIC) performs life transition health screening in the elderly at 66 years of age. Screening items include the “Timed Up and Go” (TUG) test that evaluates lower limb function by measuring walking speed, the “one-leg standing test with the eyes open” (OLS test) that evaluates balance function, and other tests that screen for cognitive impairment. The present study examined the relationship between lower limb and cognitive functions in the Korean elderly adults who underwent the life transition health screening for 66-year-olds administered by the KNHIC to investigate the usefulness of TUG and OLS tests in screening cognitive disorders and to help improve the follow-up health management and quality of life in elderly persons undergoing life transition health screening.

## 2. Materials and Methods

### 2.1. Study Subjects

Study subjects were elderly persons who underwent life transition health screening for 66-year-olds between 2010 and 2014 out of the sample registered in the National Health Insurance Service-National Sample Cohort Database (NHIS-NSC DB). Of the 34,590 individuals who underwent the health screening over the 5-year period, 4872 were excluded because they could not walk alone without assistive devices, and 29,718 were selected for the final study sample. The study was approved by the Institutional Review Board (IRB) at Kyungpook National University Hospital, which waived informed consent for the retrospective analysis using national cohort database.

### 2.2. Study Procedure

#### 2.2.1. Life Transition Health Screening for 66-Year-Olds

Life transition health screening is a national health screening program administered by the KNHIC for individuals turning 66 years of age in the year of screening. In the first stage of the health examination, a questionnaire is used to examine each individual’s disease history, family history, and health behaviors and to screen for cognitive disorders and depression. In addition, anthropometric and physical tests (height, weight, waist circumference, vision, hearing, and blood pressure), blood tests, urinalysis, chest radiography, bone density testing (for women), and lower limb function tests are performed. The second stage of health screening is conducted if any abnormality was detected on screening tests for hypertension, diabetes, cognitive impairment, or depression.

#### 2.2.2. Laboratory Tests

Blood specimens are obtained after an 8-h fasting period to measure the levels of hemoglobin (Hb), fasting blood glucose (FBG), total cholesterol (TC), high density lipoprotein cholesterol (HDL-C), low density lipoprotein cholesterol (LDL-C), creatinine, alanine aminotransferase (ALT), aspartate aminotransferase (AST), and gamma-glutamyl transferase (GGT).

#### 2.2.3. Assessment of Health Behaviors

Based on their responses to a questionnaire item regarding smoking behavior, subjects were classified as current smokers or current non-smokers; the latter included those who had smoked in the past. Alcohol consumption habits were assessed in the questionnaire in terms of the frequency and amount of alcohol consumed per week. Subjects were categorized, based on their responses to the items, according to the classification of the US Centers for Disease Control and Prevention (CDC) as follows: Non-drinker (consumes less than a glass of alcohol per week), social drinker (consumes 1–7 glasses of alcohol per week), and heavy drinker (consumes ≥8 glasses of alcohol per week). Physical activity was assessed using the World Health Organization’s (WHO) recommendation of physical activity for the elderly, namely vigorous aerobic activity for 150 min or longer per week, as the cutoff. Subjects were classified as being active if they responded “5 times or more” to the questionnaire item, “These days, for how many days a week do you perform vigorous activities for 30 min or more, such that you are more out of breath than usual (for example, power walking, playing tennis doubles, cycling at an average speed, or squatting to mop the floor)?”, and as being inactive if the frequency was less than 5 times per week.

#### 2.2.4. Assessment of Lower Limb Function

Two aspects of lower limb function were assessed: walking speed and balance function. In the TUG test used to measure walking speed, the time taken for the subject to get up from a standard armchair, walk a 3-m distance, turn around, and return to sitting in the chair, was recorded. The performance was classified as abnormal if it took longer than 10 s, following the criterion used by the KNHIC [8]. In the OLS test performed to assess balance function, the duration that the subject was able to stand on one leg with the eyes open was measured; a duration <20 s was categorized as abnormal following the KNHIC criterion [9].

#### 2.2.5. Assessment of Cognitive Function

During the first stage of health screening, cognitive function was assessed based on the responses of either the subject or a guardian who accompanied the subject to 5 questions screening for cognitive impairment (Prescreening Korean Dementia Screening Questionnaire; KDSQ-P). The 5 KDSQ-P questions were: “Do you think your memory is worse than that of your friends and colleagues?”, “Do you think your memory has deteriorated compared to a year ago?”, “Has your memory been a problem when you were doing something important?”, “Do others notice that your memory has deteriorated?”, and “Do you find that you do not perform daily activities as well as you did before?” For each question, subjects chose 1 of 3 responses: “no”, “sometimes”, and “frequently,” to which the scores of 0, 1, and 2 were assigned, respectively. Subjects whose total score was ≥4 were categorized into the high-risk group for cognitive disorders [10]. These high-risk patients underwent further cognitive function assessment in the second stage of health screening; for this, the Korean Dementia Screening Questionnaire-Cognition (KDSQ-C) was used. KDSQ-C comprises 5 items for each of the following: memory, linguistic ability, and the ability to perform a complex task, for a total of 15 items. Each item scores 0 (“not at all”), 1 (“sometimes”), or 2 (“frequently”) points, and subjects with a total score ≥ 6 points are classified as being suspected of having a cognitive disorder [11].

#### 2.2.6. Screening for Depression

A depression score was obtained based on the responses to 3 mood items included in the first-stage health screening questionnaire. The questions were selected from 15 items of the Korean version of the Short-form Geriatric Depression Scale (SGDS-K) [12]. These questions were: “Have you experienced decreased activity or motivation lately?”, “Do you feel useless at present?”, and “Do you feel that your current situation has no hope at all?” Each question was answered either “yes” (1 points) or “no” (0 points).

### 2.3. Statistical Analysis

To examine the high-risk group for cognitive disorders and the normal group classified in the first stage of health screening, independent *t*-tests and chi-squared tests were used for continuous variables and discrete variables, respectively. Additionally, the characteristics of subjects’ in the group suspected of having cognitive disorders (based on the KDSQ-C score performed in the first stage of health screening) and the normal group were examined separately. To examine the relationship between cognitive and lower limb functions, Pearson’s correlational analysis and logistic regression analysis were performed. To estimate an appropriate cutoff criterion to assess cognitive disorders, receiver operating characteristic (ROC) curve analysis was conducted; this was performed using MedCalc version 18.6.4 (MedCalc Software, Mariakerke, Belgium). All other statistical analyses were performed using IBM SPSS for Windows version 23.0 (IBM SPSS Statistics, Armonk, NY, USA). Statistical significance was set at *p* < 0.05 in all analyses. 

## 3. Results

### 3.1. Subject Characteristics According to First-Stage Cognitive Function Screening

During the first stage of health screening, 26,436 (89.0%) subjects were classified into the normal group (KDSQ-P < 4) and 3282 (11.0%) into the high-risk group for cognitive disorder (KDSQ-P ≥ 4). These groups did not differ significantly in terms of physical examination findings (height, weight, blood pressure), laboratory test results, and disease history (Table 1). The proportion of current smokers was significantly greater in the high-risk group (21.2%) than in the normal group (17.8%), and the proportion of heavy drinkers was also higher in the high-risk group. The walking speed of the group at high-risk group of cognitive disorders was significantly slower than that of the normal group, with mean TUG times of 15.00 s and 8.44 s, respectively (*p* < 0.001). Similarly, balance function was worse in the high-risk than in the normal group, with mean OLS times of 9.35 and 17.61 s, respectively (*p* < 0.001). There was a significant difference in mean depression score between the 2 groups, with scores of 4.21 in the group at high-risk of cognitive disorders and 1.56 in the normal group (*p* < 0.001). 

### 3.2. Subject Charazcteristics according to Second-Stage Cognitive Function Screening

Table 2 shows the results of the analysis conducted to compare the normal group (KDSQ-C < 6) and the group of patients suspected of having a cognitive disorder (KDSQ-C ≥ 6), classified based on the KDSQ-C scores of the 3282 subjects identified during first-stage screening as being at high risk of a cognitive disorder. Of these 3282 subjects, 655 (20.0%) were categorized into the normal group and 2627 (80.0%) into the group suspected of having a cognitive disorder. Similar to the first-stage health screening findings, the groups were not significantly different in terms of physical examination findings, laboratory test results, and disease history, but the group suspected of cognitive disorders scored significantly higher in the depression screening test (*p* < 0.001). Rates of current smokers and heavy drinkers were significantly higher in the group suspected of cognitive disorders than in the normal group (18.6% vs. 21.9%, and 12.1% vs. 16.1%, respectively; *p* < 0.001 for both). The walking speed was significantly slower in the possible cognitive disorder group than in the normal group, with mean TUG times of 15.89 s and 11.42 s, respectively (*p* < 0.001). The mean OLS time was significantly shorter in the possible cognitive disorder group than in the normal group (8.68 vs. 12.08; *p* < 0.001).

### 3.3. Relationship between Cognitive Function and Lower Limb Function

Cognitive function showed a significant positive correlation with TUG interval (*r* = 0.414, *p* < 0.001) and a significant negative correlation with OLS duration (*r* = −0.237, *p* < 0.001). A partial correlation analysis conducted after the effects of smoking, alcohol consumption, and physical activity were removed also revealed a significant positive correlation between cognitive function and TUG interval (*r* = 0.403, *p* < 0.001) and a significant negative correlation between cognitive function and OLS duration (*r* = −0.227, *p* < 0.001), as shown in Table 3.

### 3.4. Relative Risk of Cognitive Disorder according to Lower Limb Function

Table 4 shows the relative risk of a cognitive disorder in the normal group compared with abnormal groups that were determined based on a TUG interval >10 s or an OLS duration <20 s, according to the criteria used in the life transition health screening program for 66-year-olds. Model 1 was computed without adjustment. Model 2 was adjusted for smoking status, alcohol consumption status, and physical activity, and Model 3 was adjusted for the depression screening test score in addition to the variables included in Model 2. The odds ratio (OR) for cognitive impairment in the prolonged TUG interval (abnormal) group relative to the corresponding normal group was 1.21 (95% confidence interval (CI), 1.19–1.24) in Model 1, 1.25 (95% CI, 1.09–1.39) in Model 2, and 1.23 (95% CI, 1.09–1.33) in Model 3; all ORs were statistically significant. Regarding OLS duration, the OR of the abnormal group versus the corresponding normal group was statistically significant in all models: 1.09 (95% CI, 1.08–1.11) in Model 1, 1.10 (95% CI, 1.06–1.12) in Model 2, and 1.08 (95% CI, 1.03–1.14) in Model 3.

To conduct trend analyses, the variables TUG interval and OLS duration were divided into quartiles (TUG interval: Q1, ≤11 s; Q2, 12–15 s; Q3, 16–19 s; Q4, ≥20 s and OLS duration: Q1, ≥13 s; Q2, 8–12 s; Q3, 6–7 s; Q4, ≤5 s). These analyses showed that a greater proportion of subjects suspected of having a cognitive disorder fell into the upper quartiles (*p* for trend <0.001 for both) (Table 5).

The ORs showed an increasing trend in quartile groups with longer TUG intervals compared with quartile groups with shorter TUG intervals (*p* for trend < 0.001). Moreover, the OR of the uppermost quartile group relative to the lowermost quartile group was 20.15 (95% CI, 13.31–30.52) and was statistically significant. The ORs were also significant in Models 2 and 3. The OR of the group with the shortest OLS duration relative to that with the longest OLS duration was 3.47 (95% CI, 2.72–4.42) and the risk showed an increasing trend as OLS duration decreased (*p* for trend < 0.001). The ORs in models 2 and 3 were also significant (Table 6).

### 3.5. Optimal Criteria for Lower Limb Function to Screen for Cognitive Disorders

**Figure 1:**
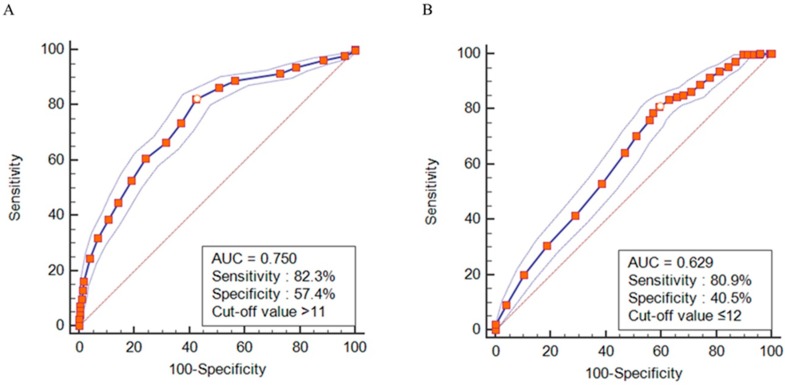
Receiver operating characteristic curve of TUG interval and OLS duration for predicting cognitive disorders. (**A**) Timed Up-to-Go test interval. (**B**) One-leg stand test duration. TUG, Timed Up-and-Go test; OLS, one-leg standing test; AUC, area under the curve.

ROC curve analysis was performed to identify appropriate cutoff values for TUG interval and OLS duration to screen for cognitive disorders. The optimal TUG interval to screen for cognitive disorder was >11 s; based on this criterion, the sensitivity was 82.3%, the specificity was 57.4%, and the area under the curve (AUC) was 0.750 (95% CI, 0.735–0.764, *p* < 0.001). The optimal OLS duration to screen for a cognitive disorder was ≤12 s; based on this cutoff criterion, the sensitivity was 80.9%, the specificity was 40.5%, and AUC was 0.629 (95% CI, 0.612–0.645, *p* < 0.001), as shown in Figure 1.

## 4. Discussion

In the present study, anthropometric measurements, laboratory test results, and disease history did not differ significantly between the group at high risk of cognitive disorder and the normal group. These findings are consistent with findings on mean anthropometric measurements, laboratory test results, and chronic disease prevalence among people in their 60s that were reported in the 2014 National Health Statistics that were based on the Korea National Health and Nutrition Examination Survey (KNHANES) and were presented by the Ministry of Health and Welfare [13]. The present study used data extracted from the NHIS-NSC DB, and, considering that the general characteristics of the study subjects were not very different from those reported in the National Health Statistics, the findings in this study can be regarded as representative of elderly Koreans undergoing national health screening at 66 years of age.

An objective of the study was to examine the relationship between lower limb function and cognitive function; the relevant findings were as follows: The high-risk group was found to have a slower walking speed and reduced balance function compared with the normal group. Cognitive function, evaluated via KDSQ-C, was significantly correlated with lower limb function, measured via TUG interval and OLS duration. Normal walking comprises initiating and maintaining regular stepping, balancing to maintain posture, and proper judgment and adaptability regarding the space occupied by the body [14]. To walk normally, all parts of the nervous system (sensory nervous systems to receive visual and proprioceptive sensations; the frontal lobe to establish motor plans; the basal ganglia in charge of automaticity; the cerebellum in charge of coordination and adaptation; the spinal cord, nerve roots, and peripheral nerves to transmit motor and neural signals; and the brainstem to integrate these signals) and the musculoskeletal system (that actually performs the motion of stepping) are required to function normally and operate in balanced way [15].

In general, gait disturbance increases with age. As age increases, walking speed decreases and it is more difficult to maintain balance, increasing the risk of fall [16]. Han et al. demonstrated that the factor showing the strongest correlation with walking speed is age [14], and Sudarsky reported that only 18% of individuals older than 85 years walk normally [17]. It may be speculated that gait disturbance with aging is most influenced by decreased muscle mass, which occurs as part of normal aging as the production of growth hormone and androgens decreases and protein consumption is reduced [18]. However, not all elderly persons experience gait disturbance. Accordingly, active research on gait disturbances in the elderly is being conducted, with particular attention focused on gait disturbance as a predictor of cognitive disorders [2].

In a positron emission tomography (PET) study, Malouin et al. showed that the areas of the brain related to cognitive function (the hippocampus, frontal lobe, prefrontal lobe, etc.) are activated during normal walking [19]. The hippocampus integrates signals from the sensory nervous system [20]. The prefrontal lobe is responsible for attention, is directly involved in walking [21], and is functionally linked to the hippocampus (hence, it may have an indirect effect on walking) [22]. In patients with diseases that are accompanied by cognitive impairment such as Alzheimer’s disease and vascular dementia, if degenerative changes occur in the areas of the brain that play an important role in maintaining cognitive function—such as the frontal lobe and hippocampus—gait disturbance (reduced walking speed and difficulty in maintaining balance) may develop. Hashimoto et al. conducted a cross-sectional study and found a significant association between hippocampus atrophy and physical inactivity [23]. According to Pettersson et al., walking speed is slowed not only in patients with advanced but also those with early Alzheimer’s disease [24]. Verghese et al. demonstrated that even individuals with mild cognitive impairment—who only experience memory decline but can perform activities of daily living normally—might demonstrate a slowed walking speed [25]. Hashimoto et al. found a significant correlation between cognitive function tests and TUG test [26]. In the present study, as in previous studies, a significant correlation was found between walking speed and cognitive function. In addition, in this study cognitive function was found to be correlated with balance function, a topic insufficiently investigated to date.

Balancing the body is achieved via the balanced workings of visual, proprioceptive, and somatosensory systems. The human body maintains balance via the ability to orient itself in space, using visual information collected through the eyes and proprioceptive sensations to recognize angular movement of the head and changes in position relative to gravity. These sensory inputs are integrated in the hippocampus [27]. Thus, the ability to maintain balance may also decrease if the hippocampal ability to integrate sensations decreases.

The TUG and OLS tests were originally used as tools to screen for the risk of falling in the life transition health screening program for 66-year-olds. TUG test was proposed by Podsiadlo et al. [8] and is widely used as a tool to assess motor ability in the elderly owing to its significance and convenience. Podsiadlo et al. defined a TUG test performance of <10 s as being normal, and Shumway-Cook et al. reported a TUG duration of ≥13.5 s as being a strong predictor of falls [28]. The OLS test is included in the Performance Oriented Mobility Assessment (POMA) developed by Tinetti et al. [9], and is reported to be useful in assessing decreased physical function in the elderly [29]. Kim et al. reported a significant difference in OLS duration between an elderly group with falls and a normal elderly group (19.7 s and 36.8 s, respectively) [30]. Similarly, Cehlsen et al. found a significant difference in OLS duration between the groups who had and had not experienced falls (10.9 s and 18.7 s, respectively) [31]. As discussed above, the TUG and OLS tests are used to assess the risk of falls in the elderly in the life transition health screening for the 66-year-olds; used for this purpose, previous research has defined a TUG interval of ≤10 s and an OLS duration of ≥20 s as being normal.

In the present study, we first determined abnormal groups based on the criteria of a TUG interval >10 s and an OLS duration <20 s, following the guidelines of the life transition health screening program for 66-year-olds; these variables were found to be significantly related to cognitive function, as evaluated using the KDSQ-C questionnaire. Further, we divided subjects into quartiles based on their TUG and OLS test results and found that the risk of cognitive disorder increased as the TUG interval increased and OLS duration decreased. Moreover, these tests were found to be useful as tools to screen for cognitive disorder in the elderly. As a screening tool, the TUG test had an AUC of 0.750, a sensitivity of 82.3%, and a specificity of 57.4% at a cutoff of ≥11 s. This finding is not largely different from the current criterion of >10 s used to screen for fall risk, and thus, TUG interval is considered to be useful in screening for cognitive disorders as well as fall risk. As a screening tool, the OLS test had an AUC of 0.629, a sensitivity of 80.9%, and a specificity of 40.5% at a cutoff of ≤12 s. This finding is different from the current criterion used to screen for fall risk. Elderly persons without cognitive disorders may manifest decreased balance function due to physical problems that occur as part of normal aging, such as reduced muscle mass; this may increase their fall risk [32]. Elderly persons with impaired cognitive function are thought to show more severe balance problems, in addition to other physical problems of aging. This is hypothesized to be the reason that the optimal cutoff value for the OLS test in the present study was lower than that used to screen for fall risk. Additional studies should be conducted, including prospective research on the usefulness of the OLS test as a screening tool for cognitive disorders. The causal relationship between fall and cognitive function should also be explored.

There are a few limitations to this study. First, between-sex differences in physical characteristics or health behaviors were not considered, because the NHIS-NSC DB (the source of data used in the present study) does not identify an individual’s sex. However, in the previous studies, there was no significant difference according to sex in the lower limb function evaluate using the TUG and OLS test [8,9]. In addition, there was no between-sex difference in the cut-off values for each tool for predicting the falls [28,29]. Considering the above, the results of this study, which do not consider sex-related differences, may be meaningful. Further studies should be conducted considering other variables including gender. Second, an individual’s history of cognitive disorder or dementia and history of taking medications are not available in the database, so it was not possible to consider these factors in the analysis. Third, the normal group determined in the first-stage health screening was not sufficiently examined, because only those classified as being at high risk for a cognitive disorder based on the KDSQ-P were further screened using the KDSQ-C in the second stage. Lastly, although we demonstrated an association between lower limb function and cognitive function, the cross-sectional design of the study precludes any inference of a causal relationship. Nonetheless, the strengths of the present study include that the findings can be generalized to the relevant population, as it was based on a representative sample by using NHIS-NSC DB, and that age—an important factor affecting both lower limb and cognitive functions—was controlled for by limiting the sample to the those aged 66 years.

## 5. Conclusions

This study showed that cognitive function is related to walking speed and balance function in relatively healthy elderly persons who were able to visit a medical center to undergo the life transition health screening program for 66-year-olds. These individuals performed the tasks of getting up to walk a distance and returning to the starting place and standing on one leg. Additionally, this study demonstrated the usefulness of the TUG and OLS tests as screening tools for cognitive disorders. Thus, simple tests lasting <1 min (as opposed to comprehensive questionnaire-based assessment for cognitive function) can be performed for the elderly during the national health screening program, in which a large number of individuals participate. This is expected to be a very useful finding for both management and therapy of the health of the elderly. In the future, a large-scale prospective cohort study should be conducted to examine changes in cognitive and lower limb functioning in the elderly, and to investigate the causal relationship between these variables.

## Figures and Tables

**Table 1:** Clinical characteristics of subjects according to primary screening tests for cognitive function.

Characteristic	Normal (KDSQ-P < 4) (*n* = 26,436)	High-Risk for Cognitive Disorder (KDSQ-P ≥ 4) (*n* = 3282)	*p*-Value
Height, cm	161.31 ± 8.72	160.99 ± 9.22	0.189
Weight, kg	63.35 ± 11.22	62.99 ± 10.51	0.259
Systolic BP, mmHg	124.45 ± 16.81	124.11 ± 15.53	0.676
Diastolic BP, mmHg	73.51 ± 11.73	73.82 ± 11.41	0.552
Fasting glucose, mg/dL	102.82 ± 10.93	103.01 ± 11.22	0.197
Total cholesterol, mg/dL	189.53 ± 35.29	189.38 ± 36.11	0.552
Triglyceride, mg/dL	140.91 ± 23.62	141.66 ± 23.52	0.094
HDL-C, mg/dL	47.53 ± 15.42	47.21 ± 15.72	0.582
LDL-C, mg/dL	113.87 ± 29.61	114.03 ± 26.81	0.134
Hemoglobin, g/dL	14.52 ± 2.51	14.31 ± 2.94	0.389
Serum creatinine, mg/dL	8.93 ± 1.31	8.89 ± 1.32	0.498
AST, U/L	26.53 ± 13.63	26.43 ± 13.61	0.586
ALT, U/L	22.61 ± 18.52	23.19 ± 18.48	0.274
GGT, U/L	25.17 ± 34.92	24.92 ± 35.69	0.267
Past history			
Hypertension	12,980 (49.1)	1625 (49.5)	0.179
Diabetes	6715 (25.4)	857 (26.1)	0.078
Dyslipidemia	7455 (28.3)	919 (28.0)	0.362
Heart disease	582 (2.2)	79 (2.4)	0.189
Tuberculosis	1137 (4.3)	138 (4.2)	0.533
Cancer (any)	2670 (10.1)	345 (10.5)	0.089
Smoking			<0.001
None	21,730 (82.2)	2586 (78.8)	
Current	4706 (17.8)	696 (21.2)	
Alcohol consumption			0.003
None	10,442 (39.5)	1168 (35.6)	
Social	13,060 (49.4)	1611 (49.1)	
Heavy	2934 (11.1)	503 (15.3)	
Physical activity			0.002
Inactive	14,090 (53.3)	1835 (55.9)	
Active	12,346 (46.7)	1447 (44.1)	
TUG interval, s	8.44 ± 3.24	15.00 ± 5.26	<0.001
OLS duration, s	17.61 ± 4.67	9.35 ± 6.06	<0.001
Depression screening score *	0.52 ± 2.76	1.40 ± 3.04	<0.001

KDSQ-P, Prescreening Korean Dementia Screening Questionnaire; BP, blood pressure; HDL-C, high density lipoprotein cholesterol; LDL-C, low density lipoprotein cholesterol; AST, aspartate aminotransferase; ALT, alanine aminotransferase; GGT, gamma-glutamyl transferase; TUG, Timed Up-and-Go test; OLS, one-leg standing test. All values are presented as the mean ± standard deviation, or number (%). * Scores were calculated based on answers to the three items in the Korean version of Short-form Geriatric Depression Scale questionnaire. *p*-values were calculated using the chi-square test for discrete outcomes and independent *t*-test for continuous outcomes.

**Table 2:** Characteristics of subjects at high-risk of cognitive disorders according to the Korean Dementia Screening Questionnaire-Cognition (KDSQ-C) test.

Characteristic	Normal (KDSQ-C < 6) (*n* = 655)	Suspected Cognitive Disorder (KDSQ-C ≥ 6) (*n* = 2627)	*p*-Value
Height, cm	161.11 ± 10.62	160.87 ± 8.10	0.169
Weight, kg	62.96 ± 11.23	63.02 ± 12.51	0.439
Systolic BP, mmHg	124.16 ± 16.10	124.06 ± 15.13	0.576
Diastolic BP, mmHg	73.69 ± 11.77	73.95 ± 12.01	0.722
Fasting glucose, mg/dL	102.99 ± 8.93	103.03 ± 9.62	0.754
Total cholesterol, mg/dL	189.33 ± 32.29	189.43 ± 34.11	0.750
Triglyceride, mg/dL	141.02 ± 21.61	142.30 ± 22.42	0.054
HDL-C, mg/dL	47.43 ± 15.22	46.99 ± 15.82	0.082
LDL-C, mg/dL	113.91 ± 23.61	114.15 ± 22.81	0.148
Hemoglobin, g/dL	14.37 ± 2.61	14.25 ± 2.64	0.169
Serum creatinine, mg/dL	8.91 ± 1.31	8.87 ± 1.33	0.699
AST, U/L	26.46 ± 13.63	26.40 ± 13.61	0.730
ALT, U/L	23.26 ± 18.12	23.12 ± 19.48	0.564
GGT, U/L	24.66 ± 32.12	25.18 ± 31.69	0.080
Past history			
Hypertension	323 (49.3)	1302 (49.6)	0.089
Diabetes	170 (26.0)	687 (26.2)	0.192
Dyslipidemia	184 (28.1)	735 (28.0)	0.462
Heart disease	15 (2.3)	64 (2.4)	0.389
Tuberculosis	28 (4.2).	110 (4.2)	0.813
Cancer (any)	68 (10.4)	277 (10.5)	0.632
Smoking			<0.001
None	533 (81.4)	2053 (78.1)	
Current	122 (18.6)	574 (21.9)	
Alcohol consumption			<0.001
None	251 (38.3)	917 (34.9)	
Social	325 (49.6)	1286 (49.0)	
Heavy	79 (12.1)	424 (16.1)	
Physical activity			<0.001
Inactive	357 (54.5)	1478 (56.3)	
Active	298 (45.5)	1149 (43.7)	
TUG interval, s	11.42 ± 4.38	15.89 ± 5.08	<0.001
OLS duration, s	12.08 ± 8.20	8.68 ± 5.18	<0.001
Depression screening score *	1.08 ± 2.44	1.72 ± 3.17	<0.001

KDSQ-C, Korean Dementia Screening Questionnaire-Cognition; BP, blood pressure; HDL-C, high density lipoprotein cholesterol; LDL-C, low density lipoprotein cholesterol; AST, aspartate aminotransferase; ALT, alanine aminotransferase; GGT, gamma-glutamyl transferase; TUG, Timed Up-and-Go test; OLS, one-leg standing test. All values are presented as the mean ± standard deviation, or number (%). * Scores were calculated based on answers to the three items in the Korean version of Short-form Geriatric Depression Scale questionnaire. *p*-values were calculated by chi-square test for discrete outcomes and independent *t*-test for continuous outcomes.

**Table 3:** Correlation analysis of KDSQ-C and TUG and OLS time.

	Univariate Pearson Correlation		Partial Correlation *	
	*r*	*p*-Value	*r*	*p*-Value
TUG time, s	0.414	<0.001	0.403	<0.001
OLS time, s	−0.237	<0.001	−0.227	<0.001

KDSQ-C, Korean Dementia Screening Questionnaire-Cognition; TUG, Timed Up-and-Go test; OLS, one-leg standing test. * Adjusted for smoking, alcohol consumption, and physical activity.

**Table 4:** The effect of lower limb function on the risk of a cognitive disorder.

	TUG Interval			OLS Duration		
	<10 s	>10 s	*p*-Value	>20 s	<20 s	*p*-Value
	(*n* = 734)	(*n* = 2548)		(*n* = 362)	(*n* = 2920)	
Model 1 *	1 (reference)	1.21 (1.19, 1.24)	<0.001	1 (reference)	1.09 (1.09, 1.11)	<0.001
Model 2 ^†^	1 (reference)	1.25 (1.09, 1.39)	0.004	1 (reference)	1.10 (1.06, 1.12)	<0.001
Model 3 ^‡^	1 (reference)	1.23 (1.09, 1.33)	0.007	1 (reference)	1.08 (1.03, 1.14)	0.012

TUG, Timed Up-and-Go test; OLS, one-leg standing test. All values are presented as the odds ratio (95% confidence interval). * Unadjusted model. ^†^ Adjusted for smoking, alcohol consumption, and physical activity. ^‡^ Adjusted for the covariates in Model 2 and the depression screening score.

**Table 5:** Proportion of subjects with suspected cognitive disorders in the TUG interval and OLS duration quartiles (*n* = 3282).

	TUG Interval						OLS Duration					
	Q1 ≤ 11 s (*n* = 841)	Q2 12–15 s (*n* = 929)	Q3 16–19 s (*n* = 838)	Q4 ≥ 20 s (*n* = 674)	*p*-Value	*p*-Value for Trend	Q1 ≥ 13 s (*n* = 768)	Q2 8–12 s (*n* = 864)	Q3 6–7 s (*n* = 725)	Q4 ≤ 5 s (*n* = 925)	*p*-Value	*p*-Value for Trend
Normal	376 (44.7)	155 (16.7)	98 (11.7)	26 (3.9)	<0.001	<0.001	265 (34.5)	136 (15.7)	132 (18.2)	122 (13.2)	<0.001	<0.001
Suspected cognitive disorder	465 (55.3)	774 (83.3)	740 (88.3)	648 (96.1)			503 (55.3)	727 (84.3)	593 (81.8)	803 (86.8)		

TUG, Timed Up-and-Go test; OLS, one-leg standing test. All values are presented as the number of patients (%) in each quartile. *p*-values were calculated using the chi-square test. *p*-values for trends were calculated using the Cochran Amitage trend test.

**Table 6:** The effect of lower limb function on the risk of cognitive disorder according to quartiles of TUG interval and OLS duration (*n* = 3282).

	TUG Interval					OLS Duration				
	Q1 ≤ 11 s (*n* = 841)	Q2 12–15 s (*n* = 929)	Q3 16–19 s (*n* = 838)	Q4 ≥ 20 s (*n* = 674)	*p*-Value for Trend	Q1 ≥ 13 s (*n* = 768)	Q2 8–12 s (*n* = 864)	Q3 6–7 s (*n* = 725)	Q4 ≤ 5 s (*n* = 925)	*p*-Value for Trend
Model 1 *	1 (reference)	4.04 (3.24, 5.03)	6.11 (4.75, 7.85)	20.15 (13.31, 30.52)	<0.001	1 (reference)	2.82 (2.23, 3.57)	2.37 (1.86, 3.01)	3.47 (2.72, 4.42)	<0.001
Model 2 ^†^	1 (reference)	3.91 (3.14, 4.82)	5.81 (4.21, 8.13)	23.52 (12.45, 34.12)	<0.001	1 (reference)	2.65 (2.01, 3.41)	2.48 (1.98, 3.09)	3.99 (2.95, 4.82)	0.002
Model 3 ^‡^	1 (reference)	4.09 (3.21, 5.10)	6.02 (4.31,7.84)	23.13 (12.56, 35.11)	<0.001	1 (reference)	2.70 (2.21, 3.87)	2.40 (1.90, 3.12)	3.52 (2.71, 4.59)	0.009

TUG, Timed Up-and-Go test; OLS, one-leg standing test. All values are presented as odds ratio (95% confidence interval). * Unadjusted model. ^†^ Adjusted for smoking, alcohol consumption, and physical activity. ^‡^ Adjusted for the covariates in Model 2 and for the depression screening score. *p*-values for trend were calculated using linear-by-linear association, in which each group was entered as a continuous variable.
